# WPF-Mamba: wavelet-based progressive multispectral fusion mamba for fine-grained microorganism detection

**DOI:** 10.3389/fmicb.2026.1783160

**Published:** 2026-03-10

**Authors:** Mingxing Li, Jinli Zhang, Yongzhe Zhang, Zihao Shan, Jian Yang, Amin Beheshti, Yuankai Qi

**Affiliations:** 1 School of Information Science and Technology, Beijing University of Technology, Beijing, China; 2 College of Computer Science, Beijing University of Technology, Beijing, China; 3 School of Computing, Macquarie University, Sydney, NSW, Australia

**Keywords:** algae detection, microorganisms detection, multispectral detection, multispectral fusion, visual state space model, wavelet transform

## Abstract

**Introduction:**

Accurate detection of environmental microorganisms is key to ecological monitoring and public health risk assessment. Multispectral imaging yields rich biochemical and structural cues, yet its practical use is hampered by inter-band spectral heterogeneity and the small, visually similar traits of microorganisms objects. These issues impair cross-band feature alignment and discriminability, thus limiting the performance of existing detection frameworks.

**Methods:**

To address these challenges, we propose a multispectral framework for fine-grained microorganisms detection named WPF-Mamba (Wavelet-Progressive Fusion Mamba). We design a novel Progressive Visual State Space Block (P-VSS Block). Built on the conventional VSS block, it integrates a Progressive Multi-Scale Feature Fusion (PMFF) unit to optimize hierarchical representations via stepwise context and semantic enhancement, improving subtle feature capture. WPF-Mamba further incorporates a Wavelet-based Multispectral Fusion (WMF) module, which fuses complementary spectral information through multi-scale wavelet decomposition and frequency-domain alignment, mitigating cross-band inconsistencies and enhancing microorganisms texture and spectral feature representation.

**Results:**

Based on the EMDS-7 dataset, we extended the sample set by constructing high-quality infrared samples with generative adversarial networks and generative large language models, thus forming the extended multispectral microorganisms detection dataset EMDS-7-MS. Evaluation results on the EMDS-7-MS dataset demonstrate that our method further improves the mAP@50 by 2.9% compared with the baseline model, which verifies the effectiveness of our proposed method in the task of multispectral microorganisms detection.

**Discussion:**

By addressing spectral misalignment and small-object representation limitations, WPF-Mamba offers a robust, generalizable approach for multispectral microorganisms detection. Specifically, its wavelet-based fusion and progressive feature refinement strategy presents a practical paradigm for multispectral fine-grained microorganisms analysis, which in turn contributes to the development of reliable, scalable environmental monitoring systems.

## 1 Introduction

Microorganisms object detection serves as a critical technical foundation for a wide range of applications, including environmental monitoring, clinical medicine (Zhang et al., 2024b), food safety regulation, and public health surveillance. Its primary objective is to achieve rapid, sensitive, and highly specific identification and quantitative analysis of object pathogenic microorganisms, such as algae and other pathogens. Among various microorganisms detection tasks, environmental microorganisms detection plays a particularly important role in aquatic ecosystem monitoring, harmful algal bloom (HAB) early warning, and aquatic product safety assurance (Zhang et al., 2023b).

Traditional microorganisms detection methods mainly rely on two categories: one is culture-based techniques and biochemical identification approaches (Amann et al., 1995; Lagier et al., 2015; Janda and Abbott, 2007; Patel, 2015); the other includes morphological observation, physicochemical detection (Moudř́ıková et al., 2016; Shao et al., 2015), and molecular biology techniques (Mackey et al., 1996; BERNHARD et al., 1995). Although these methods are relatively cost-effective, they suffer from several inherent limitations, including long detection cycles (typically 2–3 days or even several weeks), limited sensitivity, cumbersome operations, high professional thresholds, and poor applicability to unculturable or low-abundance microorganisms. Moreover, their detection results are easily affected by subjective factors. As a result, such approaches are increasingly inadequate for modern scenarios that require rapid response and real-time monitoring.

With the rapid advancement of molecular biology techniques (Zhang et al., 2024a), biosensing technologies, and artificial intelligence (AI)-assisted analytical methods—particularly deep learning (DL)—microorganisms object detection has evolved toward high-throughput, automated, and highly sensitive paradigms, giving rise to integrated multi-technology detection systems (Zhang et al., 2021). Deep learning-based image analysis models, relying on their powerful feature extraction and pattern recognition capabilities, have transformed microorganism detection from an inefficient manual-dominated process to an automated and standardized intelligent analysis process, significantly improving detection efficiency and result reliability (Zhang et al., 2025). Currently, artificial intelligence-based microorganism detection methods are mainly divided into two categories: methods based on Convolutional Neural Network (CNN) and methods based on Transformer.

Convolutional neural networks (CNNs), owing to their strong feature extraction capability, have become the dominant models for microorganisms image analysis (Ma et al., 2023). In the early stage, Isil et al. (2021) used a high-throughput portable imaging flow cytometer to capture and reconstruct lensless color holograms of continuously flowing liquids, extracted spatial and spectral features of each algal cell in the sample from the holograms, and then realized automatic microorganism identification through CNN. Ma et al. (2023) proposed a ResNet-152-based CNN model for microorganism image classification, achieving accurate recognition of 28 common microorganism species (including Chlorella, Scenedesmus, and Spirulina), with a classification accuracy of 94.5%. The Vision Meets Algae project (Zhou et al., 2023) introduced a benchmark dataset for microorganism recognition and conducted systematic evaluations of classical and state-of-the-art detection models, providing a valuable baseline for subsequent studies. To address the dual requirements of real-time performance and high accuracy in the detection of small and densely distributed phytoplankton, researchers have increasingly focused on the optimization and lightweight design of CNN-based detectors. Tao et al. (2025) proposed MobileYOLO-Cyano, which effectively integrates the YOLOv8 architecture with MobileNetV4 and introduces a novel AdaptiveChannelHead module to enhance multi-scale feature representation and channel-wise information modeling, resulting in significant performance improvements for genus-level cyanobacteria classification. Liu et al. (2022) developed an improved lightweight Algae-YOLO model that substantially reduces model parameters while maintaining detection accuracy, making it suitable for deployment in resource-constrained marine monitoring environments. In addition, Gong et al. (2023) constructed a large-scale, high-quality environmental microorganisms detection dataset containing 28,329 annotated images across 54 genera and developed an efficient real-time detection system, demonstrating the feasibility of generic microorganism detection models for ecological monitoring. Furthermore, several studies have proposed multi-object detection networks tailored to multiple algal populations, further enhancing species detection and classification performance in microscopic images (Sun et al., 2025). However, algal microorganisms exhibit substantial variability in morphology and species characteristics and are highly susceptible to environmental factors such as illumination conditions and water flow, posing significant challenges for image-based detection. CNN methods are limited by the characteristics of local receptive fields, have weak global context modeling capabilities, and insufficient adaptability to multi-scale and low-contrast microorganism objects.

With the rapid development of Vision Transformers (ViT) and their variants in the computer vision community, object detection paradigms have gradually shifted from CNN-dominated local feature modeling toward architectures that explicitly incorporate global contextual modeling, and these advances have begun to extend to microorganisms and microorganism image analysis tasks (Carion et al., 2020). Unlike CNNs, which rely on local receptive fields, Transformer-based models employ self-attention mechanisms to capture long-range dependencies and global contextual relationships, offering inherent advantages for detecting microorganisms characterized by small object sizes, dense spatial distributions, and significant morphological variability (Blanco et al., 2025). Ahn et al. (2024) developed a deep learning model for harmful algal bloom identification based on the Swin Transformer architecture, and constructed a corresponding dataset using 105 images covering 14 bloom-prone water areas from 2018 to 2023. To address the challenges of fine-grained microorganism classification, where inter-class differences are subtle and morphological features are highly similar, Li et al. (2025a) proposed HySwinFormer, a hybrid deep learning architecture that combines CNNs with Swin Transformers. This model leverages CNNs to extract local texture features while employing hierarchical window-based self-attention to model multi-scale global context. Experimental results demonstrate that HySwinFormer outperforms conventional CNN-based methods in fine-grained microorganism classification tasks. Furthermore, to overcome the limited utilization of multimodal information in cyanobacteria classification, Blanco et al. (2025) introduced a CNN-Transformer-based multimodal learning framework, in which CNNs are used for visual feature extraction and bidirectional Transformers encode auxiliary textual or semantic information to achieve cross-modal feature fusion. Experimental results indicate that this CNN-Transformer fusion strategy significantly outperforms single-modality and traditional deep learning approaches in cyanobacteria classification tasks. Nevertheless, Transformer methods have problems of high computational complexity and poor real-time performance, are sensitive to background noise, and have limited performance in small microorganism object detection and similar group differentiation tasks.

In summary, the current field of microbial object detection is predominantly characterized by methods based on Convolutional Neural Networks (CNNs) and Transformers. However, both architectures exhibit inherent limitations in microscopic multispectral scenarios: CNNs struggle to establish global spectral dependencies across channels, while the computational complexity of Transformers scales quadratically (*O*(*N*^2^)) with image resolution and spectral dimensions. This results in significant memory pressure and inference latency when processing high-dimensional microscopic data. The recently emerged State Space Models (SSMs), specifically Mamba, introduce a selective scan mechanism to achieve global modeling capabilities similar to Transformers while reducing computational complexity to a linear scale (*O*(*N*)). This efficiency enables Mamba to process high-band, high-resolution microscopic spectral sequences more effectively, capturing subtle biochemical fingerprints of microorganisms while significantly enhancing real-time monitoring performance. Nevertheless, despite Mamba's immense potential in general computer vision, its application in microbial object detection remains a research gap. Our study reveals that the original Mamba model faces challenges such as difficulty in detecting tiny-scale microbial targets and distinguishing between morphologically similar species.

More critically, all aforementioned methods rely solely on a single visible light band, which greatly restricts the algorithms' application scenarios and generalization capabilities. While multispectral object detection has made significant strides in areas such as precision agriculture (Li et al., 2025b), smart city governance (Zhang et al., 2024c), and remote sensing (Li et al., 2023), its development in microbial detection has been relatively slow. Multispectral detection at the microscopic scale aims to achieve precise identification of microbial individuals or micro-colonies through high-spatial-resolution spectral features. Research indicates that microscopic multispectral technology can capture unique “morpho-spectral fingerprints,” providing a new pathway for non-destructive detection. For instance, at the colony scale, multispectral infrared imaging or ultraviolet hyperspectral techniques enable highly specific identification and spectroscopic characterization of bacterial colonies (Le Galudec et al., 2021; Turra et al., 2021). At the single-cell scale, hyperspectral microscopic imaging, combined with deep transfer learning or Gram-staining assistance, has demonstrated exceptional identification accuracy and robustness for foodborne and clinical pathogens, such as Candida species (Patel, 2024). Nevertheless, these algorithms still face the challenge of insufficient multispectral feature fusion in microorganism detection scenarios.

To address the above problems, this paper proposes a Wavelet-based Progressive Multispectral Fusion Mamba (WPF-Mamba) model. The model includes two feature extraction branches, which are respectively used to extract the intrinsic features of microorganism under a single spectrum. Each branch embeds a Variable Scale Selection (VSS) Block with multi-scale progressive fusion; subsequently, the P3, P4, and P5 feature maps of the two branches are extracted for microorganism multispectral feature fusion, and a multispectral feature fusion method based on wavelet transform is proposed; finally, the fused features are input into the detection head to obtain the final microorganism detection results. The specific contributions of this paper are as follows:

1. Proposes a multispectral microorganism detection framework WPF-Mamba based on Mamba. This framework utilizes the advantages of Mamba model in long-sequence modeling and efficient inference to complete microorganism feature extraction under a single spectrum, and introduces a multispectral feature fusion module based on wavelet transform to achieve effective fusion of heterogeneous multispectral microorganism features. To the best of the authors' knowledge, this is the first multispectral object detection algorithm framework for microorganism detection.
2. Aiming at the core problems of diverse scales and similar features of microorganism objects, a new feature extraction module–Progressive Visual State Space (P-VSS) Block–is designed based on the Mamba model. Through the multi-scale progressive feature fusion mechanism, this module enhances the feature representation ability for microorganism objects of different scales and improves the differentiation accuracy of microorganism of similar groups.
3. Proposes a multispectral microorganism feature fusion module based on wavelet transform. This module can efficiently mine the complementary information of microorganism between different spectral channels, realize deep fusion of heterogeneous multispectral microorganism features while suppressing noise interference, effectively solve the problems of feature redundancy and insufficient fusion existing in traditional multispectral fusion methods, and significantly improve the detection performance of microorganism objects in complex scenarios.
4. Constructs the first multispectral dataset EMDS-7-MS for microorganism detection. This dataset is extended based on the existing visible light microorganism detection dataset EMDS-7, covering various common microorganism categories and complex scenarios such as different light conditions and background interferences, providing high-quality data support for the training and performance evaluation of multispectral microorganism detection algorithms (such as the WPF-Mamba proposed in this paper).

## 2 Materials and methods

**Figure 1:**
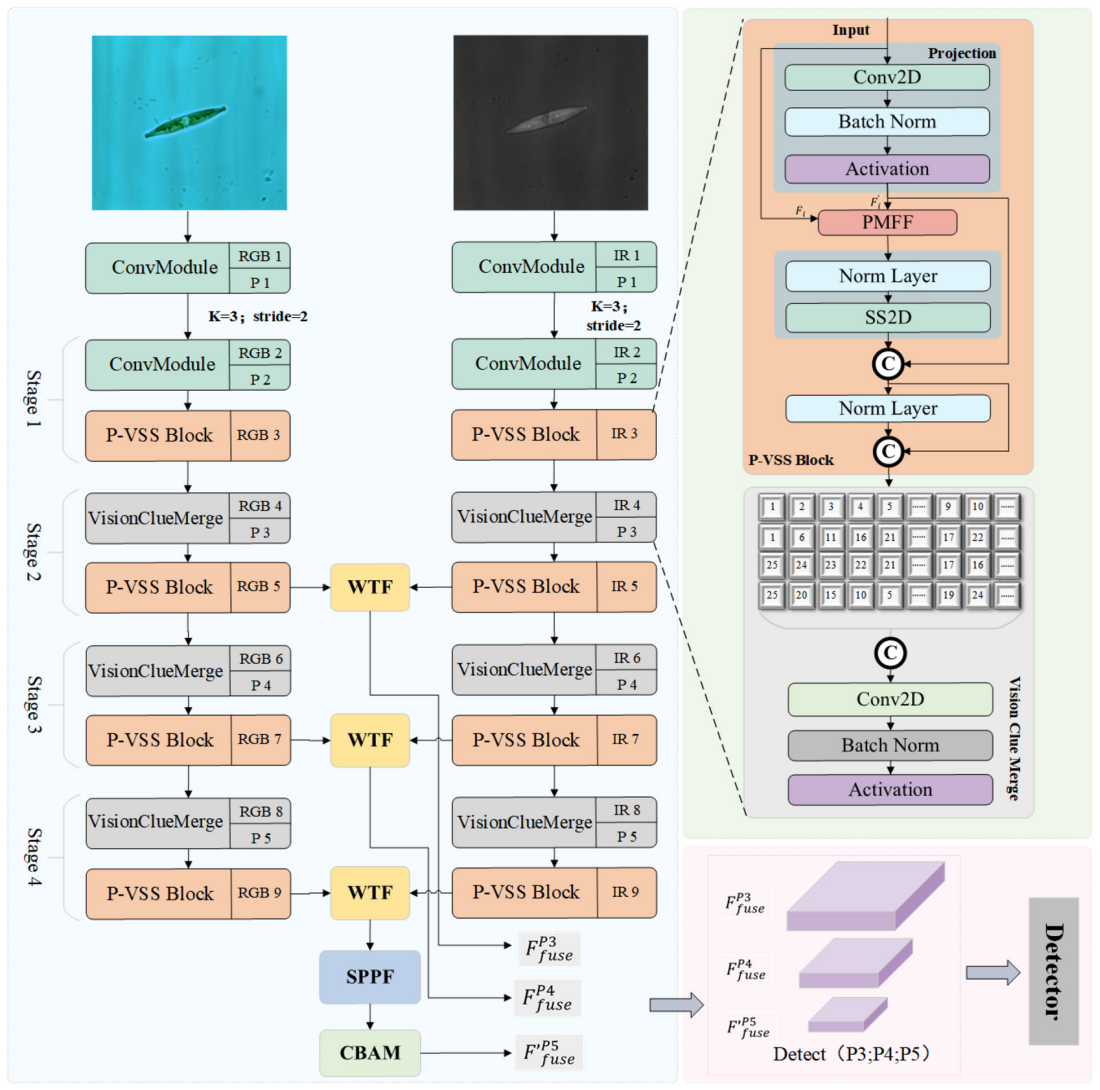
Overview of our proposed WPF-Mamba model. Each stage of the dual-branch feature extraction module contains a P-VSS Block, which performs progressive feature fusion to maintain the representational capability of object features. Meanwhile, the Vision Clue Merge module (Wang et al., 2025) is adopted for feature downsampling to reduce the size of feature maps. Finally, the multi-scale features extracted from each branch are fused through the WTF module, and the fused features of different scales are fed into the detection head to obtain the final detection results.

This chapter elaborates on the overall architecture of the proposed method and the specific design details of each sub-module. Aiming at the microorganism multispectral object detection task, this paper proposes a detection network based on YOLO-Mamba (Wang et al., 2025). The method takes YOLO-Mamba as the benchmark framework and selects three multi-scale features (P3, P4, P5) as the core processing features, with the overall architecture shown in Figure 1. The inputs of the network are visible light images and infrared images, where the visible light image is denoted as $I_{rgb}\in {\mathbb{R}}^{H\times W\times C}$ and the infrared image is denoted as $I_{ir}\in {\mathbb{R}}^{H\times W\times C}$ (where *H*, *W*, and *C* represent the height, width, and number of channels of the image, respectively). The two types of input images are fed into independent spectral feature extraction branches for single-spectrum feature learning, and the core feature extraction module of each branch is the P-VSS Block designed in this paper. After completing the single-spectrum feature extraction, the P3, P4, and P5 scale features *F*_*rgb*−*P*3_, *F*_*rgb*−*P*4_, *F*_*rgb*−*P*5_ and *F*_*ir*−*P*3_, *F*_*ir*−*P*4_, *F*_*ir*−*P*5_ output by the two branches are input into the WMF module for multispectral feature fusion, obtaining fused features *F*_*fusion*−*P*3_, *F*_*fusion*−*P*4_, *F*_*fusion*−*P*5_. Finally, the fused features pass through the detection head to complete object classification and bounding box regression, and output the detection results of microorganism objects.

### 2.1 Preliminaries

This section establishes the theoretical framework by revisiting State Space Models (SSM) (Gu et al., 2021), specifically the Mamba architecture , which serves as the cornerstone for achieving linear computational complexity. We further extend this discussion to the operational logic of VMamba (Liu et al., 2024) in the context of visual tasks.

#### 2.1.1 Fundamentals of SSM

Fundamental SSMs function by projecting a one-dimensional continuous input signal *x*(*t*) ∈ ℝ into a latent representation *h*(*t*) ∈ ℝ^*N*^ before generating the output *y*(*t*) ∈ ℝ. This dynamical system is typically characterized by the following linear ordinary differential equations (ODEs):


$$\begin{array}{l} \begin{array}{l} h^{\prime }\left(t\right)=\text{A}h\left(t\right)+\text{B}x\left(t\right),\\ y\left(t\right)=\text{C}h\left(t\right). \end{array} \end{array}$$ (1)

In this formulation, **A** ∈ ℝ^*N*×*N*^ denotes the evolution matrix, while **B** ∈ ℝ^*N*×1^ and **C** ∈ ℝ^1 × *N*^ represent the input and output projection weights, respectively.

#### 2.1.2 Selective scanning and discretization

Mamba (Gu and Dao, 2024) advances the standard SSM by incorporating a selective mechanism, allowing **A** and **B** to be parameterized as functions of the input, thereby enhancing the model's representational capacity. To adapt the continuous system for discrete sequence processing, a time-scale parameter Δ is employed to transform the matrices via the zero-order hold (ZOH) rule:


$$\begin{array}{l} \begin{array}{l} \bar{\text{A}}=\exp\left(\text{Δ}\text{A}\right),\\ \bar{\text{B}}={\left(\text{Δ}\text{A}\right)}^{-1}\left(\exp\left(\text{Δ}\text{A}\right)-\text{I}\right)\cdot \text{Δ}\text{B}. \end{array} \end{array}$$ (2)

Following discretization, the system is implemented through the following recursive mapping:


$$\begin{array}{l} \begin{array}{l} h_{k}=\bar{\text{A}}h_{k-1}+\bar{\text{B}}x_{k},\\ y_{k}=\text{C}h_{k}. \end{array} \end{array}$$ (3)

### 2.2 Progressive variable scale selection block

**Figure 2:**
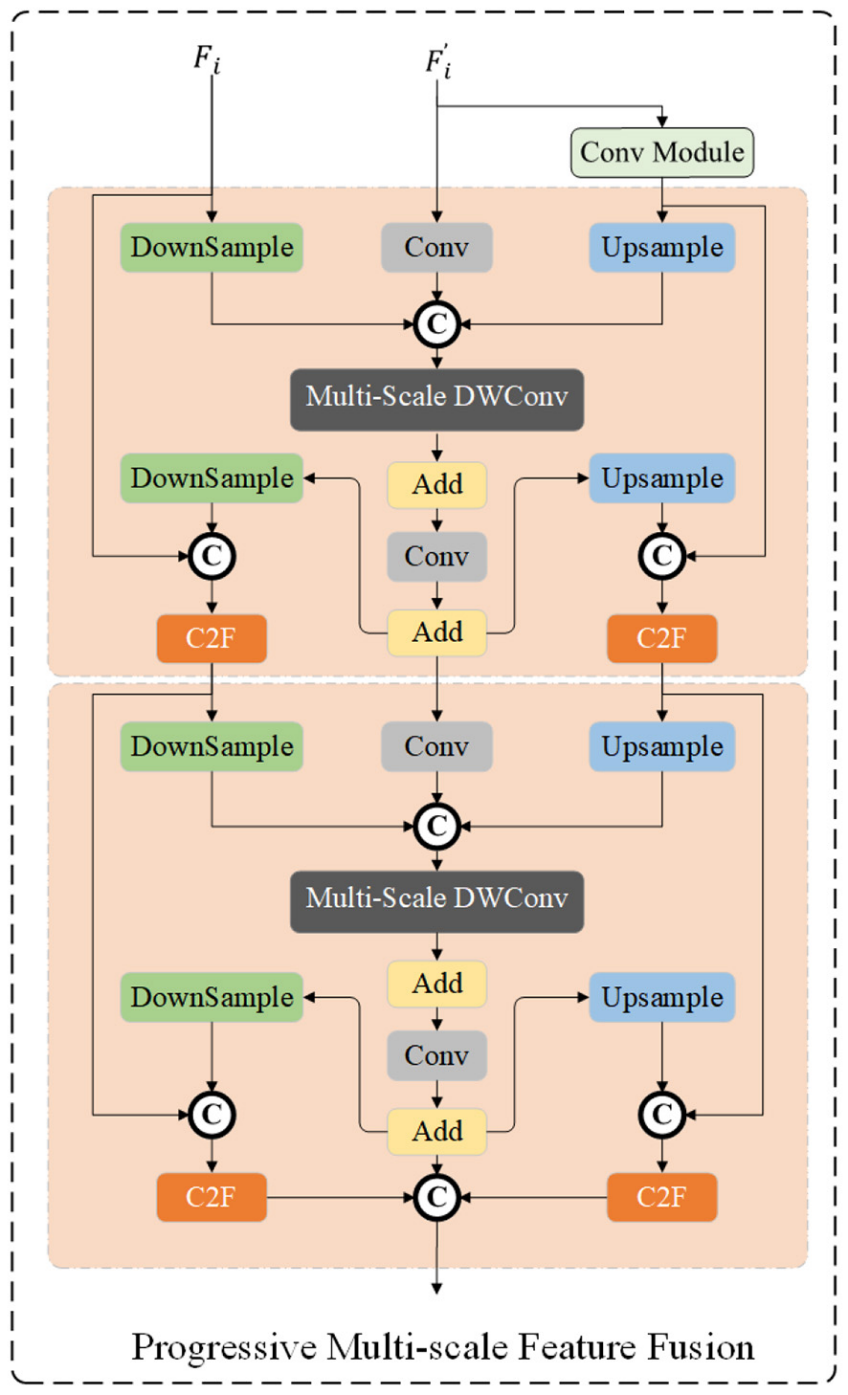
The architecture of the Progressive Multi-scale Feature Fusion (PMFF) module.

The original Mamba model suffers from feature loss when processing small-scale objects. To address this defect, this paper proposes the P-VSS Block (Progressive Variable Scale Selection Block), which incorporates the Progressive Multi-scale Feature Fusion (PMFF) module. The specific structure of the P-VSS Block is shown in Figure 2. It takes the feature *F*_*i*_ (*i* ∈ {*ir, rgb*}) of any spectral band as input, and first performs feature mapping through the Projection layer to obtain the feature $F_{i}^{\prime }$. The corresponding mathematical expression is shown in Equation 4:


$$\begin{array}{l} F_{i}^{\prime }=\text{ReLU}\left(\text{BatchNorm}\left(\text{Conv}\left(F_{i}\right)\right)\right),\;i\in \left\{ir,rgb\right\} \end{array}$$ (4)

**Figure 3:**
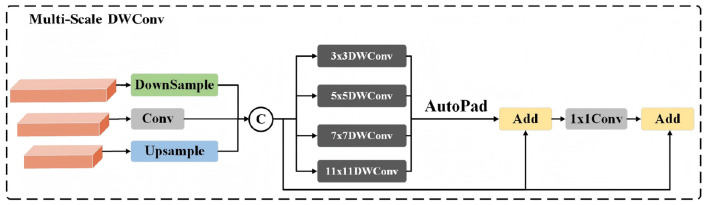
The architecture of the multi-scale feature fusion module based on DWConv.

#### 2.2.1 Progressive multi-scale feature fusion

The feature map $F_{i}^{\prime }$ is fed into the Progressive Multi-scale Feature Fusion (PMFF) module to further enhance its multi-scale representation capability. The overall architecture of PMFF is illustrated in Figure 2. The core idea of PMFF is to model the input features through parallel multi-scale wavelet-based convolutions and to achieve effective feature enhancement via a residual fusion mechanism.

#### 2.2.2 Multi-scale feature extraction via DWConv

To capture spatial-frequency information under diverse receptive fields, depth-wise separable convolution (DWConv) operations with different kernel sizes are first applied to the input feature $F_{i}^{\prime }$. This process enables the module to extract features at multiple scales simultaneously, and it can be formulated as:


$$\begin{array}{l} F_{i}^{\prime \;k}={\mathrm{DWConv}}_{k}(F_{i}^{\prime }),\;k\in \left\{5,7,9,11\right\}, \end{array}$$ (5)

where *k* denotes the kernel size of DWConv, corresponding to feature modeling branches at different scales. Since multi-scale convolution operations may introduce discrepancies in spatial resolution, an adaptive padding (*autopad*) operation is subsequently employed to spatially align the features from different scales. The aligned feature maps are then aggregated through element-wise summation to obtain the multi-scale enhanced representation:


$$\begin{array}{l} F_{i}^{\text{Multi}-\text{Scale}}=\underset{k\in \left\{5,7,9,11\right\}}{\sum }\mathrm{autopad}(F_{i}^{\prime \;k}). \end{array}$$ (6)

Finally, a residual connection combined with a convolutional transformation is introduced to fuse the original feature with the multi-scale enhanced feature, yielding the output of the PMFF module:


$$\begin{array}{l} F_{i}^{\text{PMFF}}=F_{i}^{\prime }+\mathrm{Conv}\left(F_{i}^{\prime }+F_{i}^{\text{Multi}-\text{Scale}}\right). \end{array}$$ (7)

After obtaining the output feature $F_{i}^{\text{PMFF}}$ from the PMFF module, a normalization layer followed by a two-dimensional state space modeling module (SS2D) is further introduced to capture global dependencies and enhance dynamic contextual information. This process can be formulated as:


$$\begin{array}{l} F_{i}^{\text{PMFF}-\text{SS}}=F_{i}^{\prime }⊕SS2D\left(\text{Norm}\left(F_{i}^{\text{PMFF}}\right)\right) \end{array}$$ (8)

After obtaining the feature enhanced by the PMFF module and state-space modeling (denoted as $F_{i}^{\text{PMFF}-\text{SS}}$), an additional normalization layer is first applied to stabilize the feature distribution, facilitating subsequent fusion. Subsequently, the normalized feature is concatenated with the output feature of the PMFF module ($F_{i}^{\text{PMFF}}$) along the channel dimension. Finally, the final output $F_{i}^{\text{P}-\text{VSS}}$ is obtained via the Vision Clue Merge Module (Wang et al., 2025).

### 2.3 Wavelet-based multispectral fusion module

**Figure 4:**
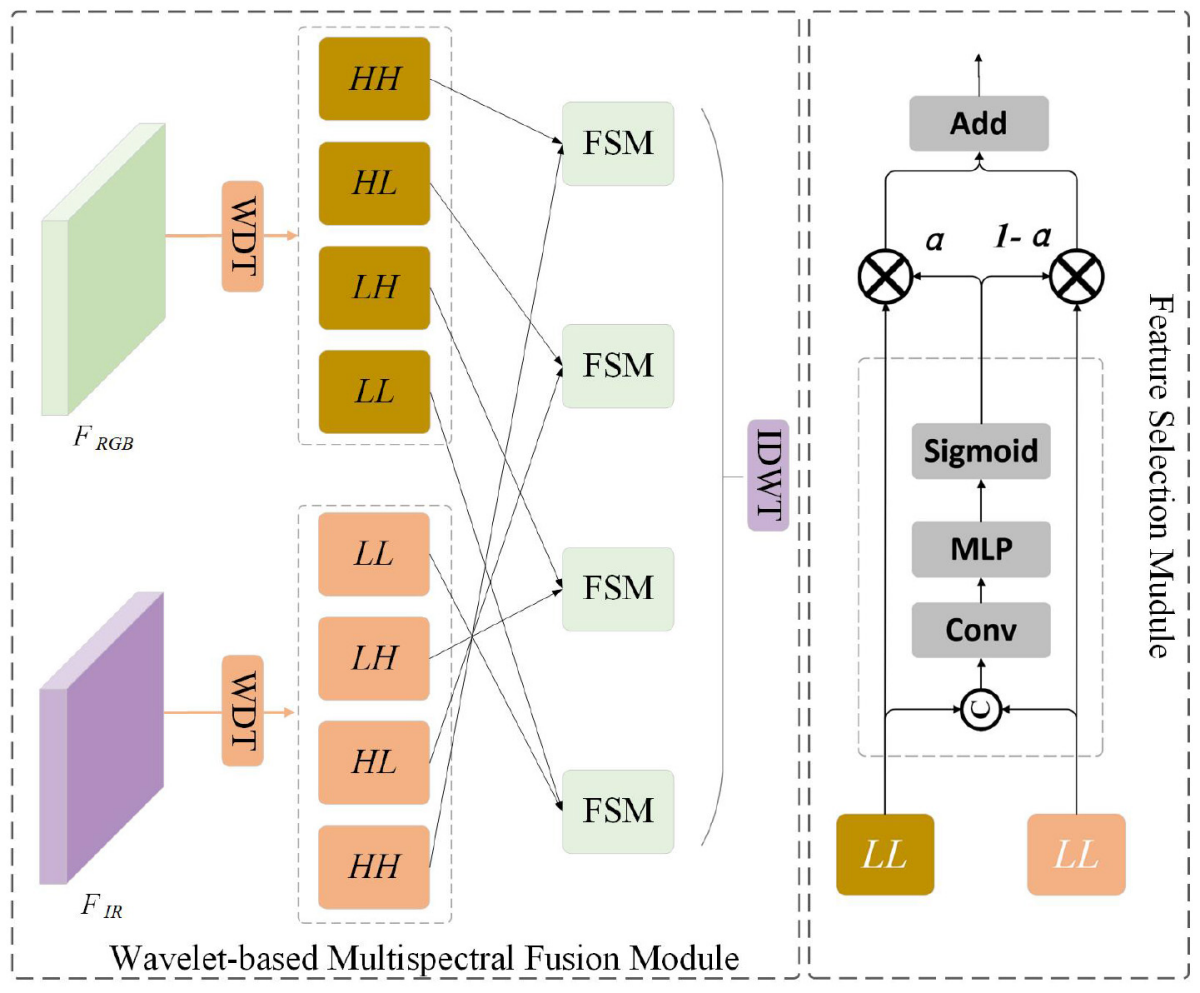
Overwrite the WMF module structure diagram.

To mitigate the challenges of spectral heterogeneity and significant noise interference in microorganism detection, we propose the Wavelet-based Multispectral Fusion (WMF) module, which leverages the Haar wavelet transform for multi-scale spectral feature decomposition. The architectural diagram of the proposed model is illustrated in Figure 4. Unlike spatial-domain fusion, the WMF module leverages the multi-resolution analysis capability of the Discrete Wavelet Transform (DWT) (Peng et al., 2025) to decouple multispectral features into distinct frequency bands. This enables the adaptive integration of high-frequency textural nuances and low-frequency structural semantics while effectively suppressing modality-specific noise.The execution logic of the WMF module follows a “decomposition-fusion-reconstruction” paradigm. Initially, dual-source spectral features–RGB light ($F_{RGB}\in {\mathbb{R}}^{C\times H\times W}$) and near-infrared ($F_{IR}\in {\mathbb{R}}^{C\times H\times W}$)—are decomposed into multi-level frequency components. Subsequently, specialized fusion strategies are applied to the low-frequency and high-frequency streams to maximize information complementarity. Finally, the Inverse Discrete Wavelet Transform (IDWT) is employed to synthesize the unified feature map *F*_*fusion*_.

#### 2.3.1 Multi-scale wavelet decomposition

We utilize the 2D-DWT to decompose each input feature *F*_*s*_ (*s* ∈ {RGB, IR}) and generate wavelet representations at different scales. Specifically, the DWT is implemented by convolving *F*_*s*_ with four distinct filters: LL_*s*_, LH_*s*_, HL_*s*_, and HH_*s*_ (*s* ∈ {RGB, IR}) each of size $\frac{H}{2}\times \frac{W}{2}\times C$. These filters correspond to the Low-Low (LL), Low-High (LH), High-Low (HL), and High-High (HH) frequency subbands, respectively. This decomposition process can be mathematically expressed as:


$$\begin{array}{l} LL_{s},HH_{s},HL_{s},LH_{s}=\text{DWT}\left(F_{s}\right) \end{array}$$ (9)

#### 2.3.2 Feature Selection Module

To address the divergent characteristics of frequency components, we implement a Feature Selection Module(FSM). The fused approximation $F_{\text{fusion}}^{i}$, where *i* ∈ {LL_*s*_, LH_*s*_, HL_*s*_, HH_*s*_} is computed as:


$$\begin{array}{l} F_{\text{cat}}^{i}=F_{\text{RGB}}^{i}⊕F_{\text{IR}}^{i} \end{array}$$ (10)

$$\begin{array}{l} \alpha =\text{Sigmoid}\left(\text{MLP}\left(\text{GAP}\left(F_{\text{cat}}^{i}\right)\right)\right) \end{array}$$ (11)

$$\begin{array}{l} F_{\text{fusion}}^{i}=\alpha \cdot F_{\text{RGB}}^{i}+\left(1-\alpha \right)\cdot F_{\text{IR}}^{i} \end{array}$$ (12)

where ⊕ denotes concatenation, GAP is global average pooling, and the learned coefficient α ensures that the more discriminative spectral band dominates the structural backbone.

#### 2.3.3 Feature reconstruction

The final fused representation is reconstructed by aggregating the processed frequency bands via 2D-IDWT:


$$\begin{array}{l} F_{\text{final}}=\text{IDWT}\left(F_{\text{fusion}}^{{\text{LL}}_{s}},F_{\text{fusion}}^{{\text{LH}}_{s}},F_{\text{fusion}}^{{\text{HL}}_{s}},F_{\text{fusion}}^{{\text{HH}}_{s}}\right) \end{array}$$ (13)

The resulting $F_{\text{final}}\in {\mathbb{R}}^{C\times H\times W}$ maintains high-fidelity spatial resolution and enriched spectral discriminability, facilitating the precise localization of fine-grained microorganisms objects.

## 3 Experiments

### 3.1 Datasets

#### 3.1.1 EMDS-7

**Figure 5:**
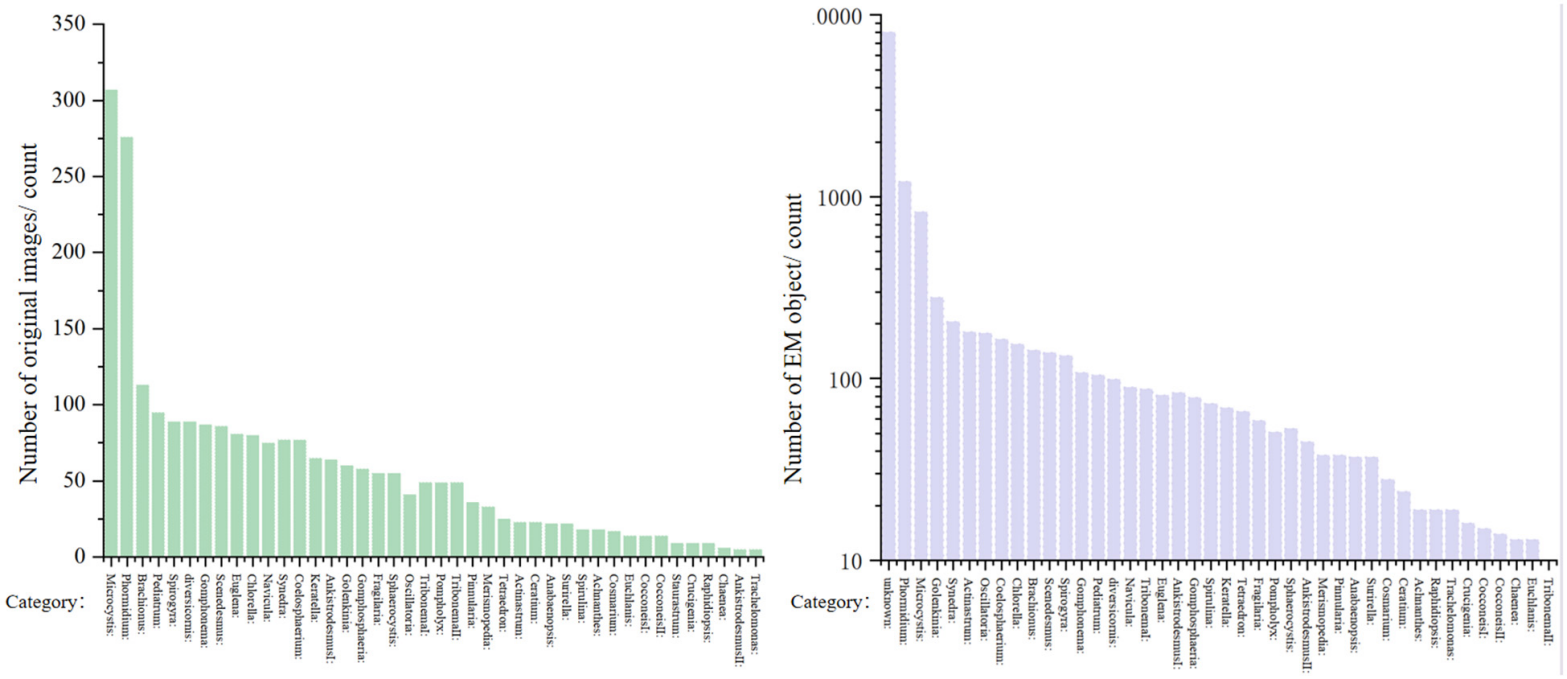
The distribution of the number of various original images and object in the EMDS-7 dataset.

This dataset is designed for environmental microorganisms detection, encompassing 41 microorganisms taxa including algae and rotifers. The original dataset had several issues, which were comprehensively reorganized and adjusted in this study. The adjustments included merging the duplicated taxon Ankistrodesmus, and revising certain annotation errors (e.g., data entry EMDS7-G011-058-0400). The specific data distribution of the revised dataset is illustrated in Figure 5. To ensure that the training, validation, and test sets all cover 41 object taxa, the dataset was partitioned at a ratio of 6:2:2 (Yang et al., 2023).

#### 3.1.2 EMDS-7-MS

**Figure 6:**
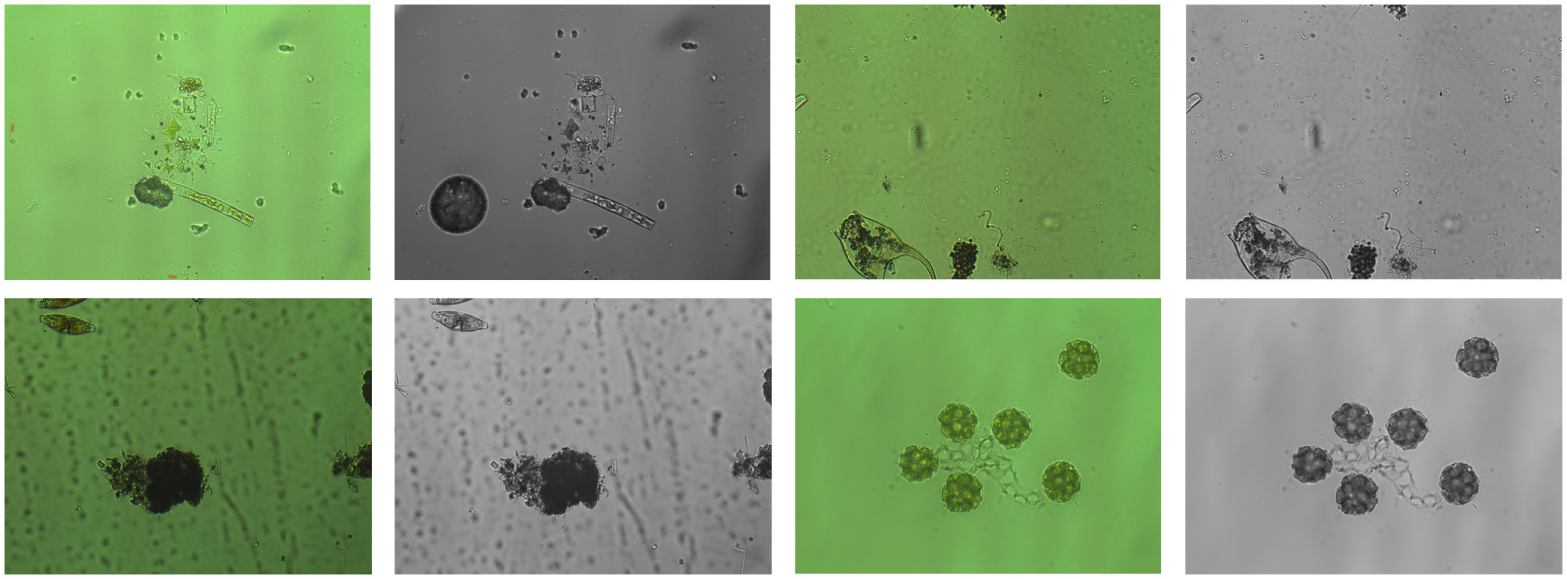
The EMDS-7-MS dataset sample includes one RGB image and one IR image.

Limited by current hardware technology constraints, the research field lacks multispectral datasets for microorganisms detection, which has consequently hindered the development of multispectral technology in this domain. To address this research gap, we generated a batch of corresponding infrared images based on the EMDS-7 dataset by leveraging infrared image generation algorithms (Ran et al., 2025) and large language models (LLMs) (Team et al., 2023). These generated infrared spectral images were then combined with the original dataset to construct a novel multispectral dataset for microorganisms algae detection, namely EMDS-7-MS. The dataset partitioning strategy is kept consistent with that of the original EMDS-7 dataset. Specific dataset samples are illustrated in Figure 6.

Because the annotations of the unknown category in the EMDS-7 dataset do not comprehensively cover all unknown object types, the unknown class is used only for training purposes and is excluded from the final evaluation of detection performance metrics.

### 3.2 Implementation details

We conducted our experiments using an Nvidia RTX 4090 GPU. We implemented our model architecture with PyTorch 2.3.1 and CUDA 12.2. For the EMDS-7-MS dataset and, the input image size is 640 × 640. The batch size for both datasets is set to 16, and the training is carried out for 100 epochs. We employed Stochastic Gradient Descent (SGD) as the optimizer, with a momentum of 0.937 and a weight decay of 0.002. We will release our code. In the testing phase, the batch size is set to 1, and the confidence threshold for visualized results is set to 0.5.

### 3.3 Baseline

- **YOLOv8-Multi** (Jocher, 2023): An extension of the YOLOv8 architecture optimized for multispectral object detection in complex scenarios.
- **SuperYOLO** (Zhang et al., 2023a): A high-resolution object detection framework that integrates super-resolution assisted training to improve the detection of small objects in remote sensing imagery.
- **CALNet** (He et al., 2023): A cross-view alignment and localization network specifically designed for multispectral object detection through effective feature fusion.
- **MDPMFN** (Liu et al., 2025): A multimodal deep progressive multi-scale fusion network that leverages diverse feature representations for robust recognition.
- **DMM** (Zhou et al., 2025): A state-of-the-art architecture based on the Selective Scanning Mechanism (Mamba), designed for efficient global dependency modeling with linear computational complexity.

### 3.4 Evaluation metrics

To quantitatively assess the performance of the proposed model, we employ Mean Average Precision (mAP) and AP@50 as the primary evaluation metrics. These metrics provide a comprehensive measure of the model's ability to accurately localize and classify objects.

The foundation of these metrics lies in Precision and Recall. Precision measures the proportion of true positive detections among all positive predictions, while Recall measures the proportion of true positives correctly identified out of all ground truth instances.


$$\begin{array}{l} \text{Precision}=\frac{TP}{TP+FP} \end{array}$$ (14)

$$\begin{array}{l} \text{Recall}=\frac{TP}{TP+FN} \end{array}$$ (15)

where *TP*, *FP*, and *FN* represent True Positives, False Positives, and False Negatives, respectively.

Average Precision (AP) summarizes the Precision-Recall curve by calculating the area under the curve. It is defined as:


$$\begin{array}{l} AP=\int_{0}^{1}p\left(r\right)dr \end{array}$$ (16)

In practical implementation, specifically for datasets like COCO or VOC, the numerical integration is often approximated using a 101-point or 11-point interpolation.

AP@50 refers to the Average Precision calculated at a fixed Intersection over Union (IoU) threshold of 0.50. IoU measures the overlap between the predicted bounding box (*B*_*p*_) and the ground truth box (*B*_*g*_):


$$\begin{array}{l} \text{IoU}=\frac{\text{Area}\left(B_{p}\cap B_{g}\right)}{\text{Area}\left(B_{p}\cup B_{g}\right)} \end{array}$$ (17)

A detection is considered a *TP* if *IoU*≥0.50; otherwise, it is classified as an *FP*.

The Mean Average Precision (mAP) is the primary metric for multi-class detection tasks. It is calculated by taking the average of AP values across all *N* categories:


$$\begin{array}{l} mAP=\frac{1}{N}\overset{N}{\underset{i=1}{\sum }}AP_{i} \end{array}$$ (18)

where *N* is the total number of classes (in your case, *N* = 41 according to the categories G001-G041) and *AP*_*i*_ is the average precision for the *i*-th class.

## 4 Result

### 4.1 Results of the EMDS-7-MS

Table 1 presents the comparison results of the AP@50 metric between the proposed method and various competing methods. The comparative methods include our baseline model YOLOv8-Multi, Super YOLO, CALNet, and MDPMFN that extract features based on convolutional neural networks, as well as DMM based on the Mamba model. Based on this environmental microorganisms detection dataset, this paper systematically evaluates the detection performance of the proposed model (Ours) on 40 microorganisms categories (after merging the duplicate category Ankistrodesmus) with AP@50 as the core evaluation metric. Experimental results demonstrate that the proposed method significantly outperforms existing multispectral object detection methods, exhibiting superior overall detection capability and remarkable performance advantages in most categories. From the perspective of the global mAP metric, the proposed model achieves an mAP of 85.6%, outperforming all comparative models. Notably, while both the DMM model and the proposed method are constructed based on the Mamba architecture, our method exceeds DMM by 2.4%, demonstrating the efficacy of our structural optimizations. Compared with CALNet, which exhibits the lowest mAP (82.3%), the proposed method achieves an improvement of 3.3%, verifying its comprehensive superiority in multi-category microorganism detection.

**Table 1:** Comparison of AP@50 across different categories (codes) for various models (the highest value in each row is bolded).

**Categories (codes)**	**YOLOV8-Multi**	**SuperYOLO**	**CALNet**	**MDPMFN**	**DMM**	**Ours**
Oscillatoria(G001)	58.2	55.7	52.3	54.9	59.1	**60.5**
Ankistrodesmus(G002&G011)	**74.4**	68.0	65.2	73.1	68.3	67.4
Microcystis(G003)	**100**	**100**	**100**	**100**	**100**	**100**
Gomphonema(G004)	79.1	68.5	66.2	78.3	77.9	**83.1**
Sphaerocystis(G005)	78.8	79.0	75.9	78.0	78.2	**82.8**
Cosmarium (G006)	**100**	**100**	**100**	**100**	**100**	**100**
Cocconeis(G007)	69.7	70.3	66.8	69.2	69.0	**73.5**
Tribonema(G008)	77.5	76.9	57.0	46.5	**84.3**	81.2
Chlorella(G009)	**100**	**100**	**100**	**100**	**100**	**100**
Tetraedron(G010)	**100**	**100**	**100**	**100**	**100**	**100**
Brachionus(G012)	**100**	**100**	**100**	**100**	**100**	**100**
Chaenea(G013)	38.3	58.9	71.2	57.8	69.1	**72.6**
Pediastrum (G014)	**100**	**100**	**100**	**100**	**100**	**100**
Spirulina(G015)	67.8	67.2	74.6	67.2	77.3	**78.5**
Actinastrum(G016)	80.1	80.8	77.3	80.2	**84.6**	83.9
Navicula(G017)	79.5	79.9	76.4	79.0	78.8	**83.2**
Scenedesmus(G018)	78.4	78.0	75.3	77.6	77.8	**82.0**
Golenkinia(G019)	77.2	76.5	75.0	76.2	76.7	**80.9**
Pinnularia(G020)	**100**	**100**	**100**	**100**	**100**	**100**
Staurastrum(G021)	32.8	77.7	**81.7**	65.5	47.0	39.9
Phormidium(G022)	76.3	77.9	75.2	72.5	79.7	**81.8**
Fragilaria(G023)	**100**	**100**	**100**	**100**	**100**	**100**
Anabaenopsis(G024)	57.9	57.4	54.8	57.1	57.5	**61.6**
Coelosphaerium(G025)	72.5	63.2	69.6	72.9	71.8	**75.9**
Crucigenia(G026)	**100**	**100**	**100**	**100**	**100**	**100**
Achnanthes(G027)	80.4	81.0	77.8	80.7	80.2	**82.4**
Synedra(G028)	78.7	78.3	75.6	77.9	78.0	**80.2**
Ceratium(G029)	81.8	82.5	79.0	82.1	81.2	**85.5**
Pompholyx(G030)	77.6	77.0	74.5	76.8	77.2	**81.3**
Merismopedia(G031)	**100**	**100**	**100**	**100**	**100**	**100**
Spirogyra(G032)	**78.1**	44.2	67.3	74.0	45.1	77.4
Coelastrum(G033)	81.1	81.9	78.3	81.5	80.7	**85.0**
Raphidiopsis(G034)	77.3	76.8	74.2	76.4	76.9	**81.0**
Gomphosphaeria(G035)	**100**	**100**	**100**	**100**	**100**	**100**
Euglena(G036)	**100**	**100**	**100**	**100**	**100**	**100**
Euchlanis(G037)	**100**	**100**	**100**	**100**	**100**	**100**
Keratella(G038)	**100**	**100**	**100**	**100**	**100**	**100**
Diversicornis(G039)	97.6	98.3	99.0	**99.9**	98.4	99.8
Surirella(G040)	78.5	78.1	75.4	77.7	77.9	**82.1**
Characium(G041)	77.7	77.1	74.7	76.9	77.1	**81.5**
mAP	82.7	82.8	82.3	82.7	83.2	**85.6**

In terms of individual category performance, 12 taxa, including Microcystis (G003) and Cosmarium (G006), achieved a saturated AP@50 of 100% across all models due to their highly distinct morphological features. The proposed method yielded the optimal AP@50 values for 25 categories (including those tied for first). Remarkable advantages were observed in categories such as Ceratium (G029) and Coelastrum (G033), where our method outperformed the second-ranked models by 3.4% and 3.1% respectively, demonstrating strong adaptability.

The proposed method failed to attain the best performance in only a few specific categories. For Staurastrum (G021), characterized by complex morphology and potentially insufficient sample representation, CALNet led with 81.7%, significantly higher than our 39.9%. In the Tribonema (G008) and Diversicornis (G039) categories, DMM and MDPMFN achieved slight leads, yet the performance gap remained narrow, with our model maintaining high detection accuracy within 3.1% and 0.1% of the top values, respectively.

### 4.2 Ablation study

The quantitative results in Table 2 validate the efficacy of each proposed component. Specifically, integrating the Mamba block into the baseline yields a 0.8% mAP boost, establishing a robust feature extraction foundation. The inclusion of PMFF (Method 2) and WTF (Method 3) further improves the performance to 85.1% and 84.3% respectively, demonstrating that progressive feature fusion and frequency-domain refinement are critical for capturing complementary multi-spectral information. Ultimately, our WPF-Mamba model achieves a peak mAP@50 of 85.6%, outperforming the baseline by 2.9%. Although the total parameters and GFLOPs increase to 20.4M and 46.5, the substantial accuracy gains justify the computational investment for high-precision multi-spectral object detection.

**Table 2:** Ablation study of different components on the multi-spectral YOLOv8n model.

**Methods**	**Mamba**	**PMFF**	**WTF**	**mAP@50 (%)**	**Parameter**	**GFLOPs**
Base	×	×	×	82.7	3.9M	11.5
Method 1	✓	×	×	83.5	14.5M	32.6
Method 2	✓	✓	×	85.1	18.2M	41.8
Method 3	✓	×	✓	84.3	16.7M	37.4
WPF-Mamba	✓	✓	✓	**85.6**	**20.4M**	**46.5**

The base model is the YOLOv8n-Multi. PMFF, and WTF represent our proposed modules. The bold values indicates the highest numerical performance.

### 4.3 Results visualization

**Figure 7:**
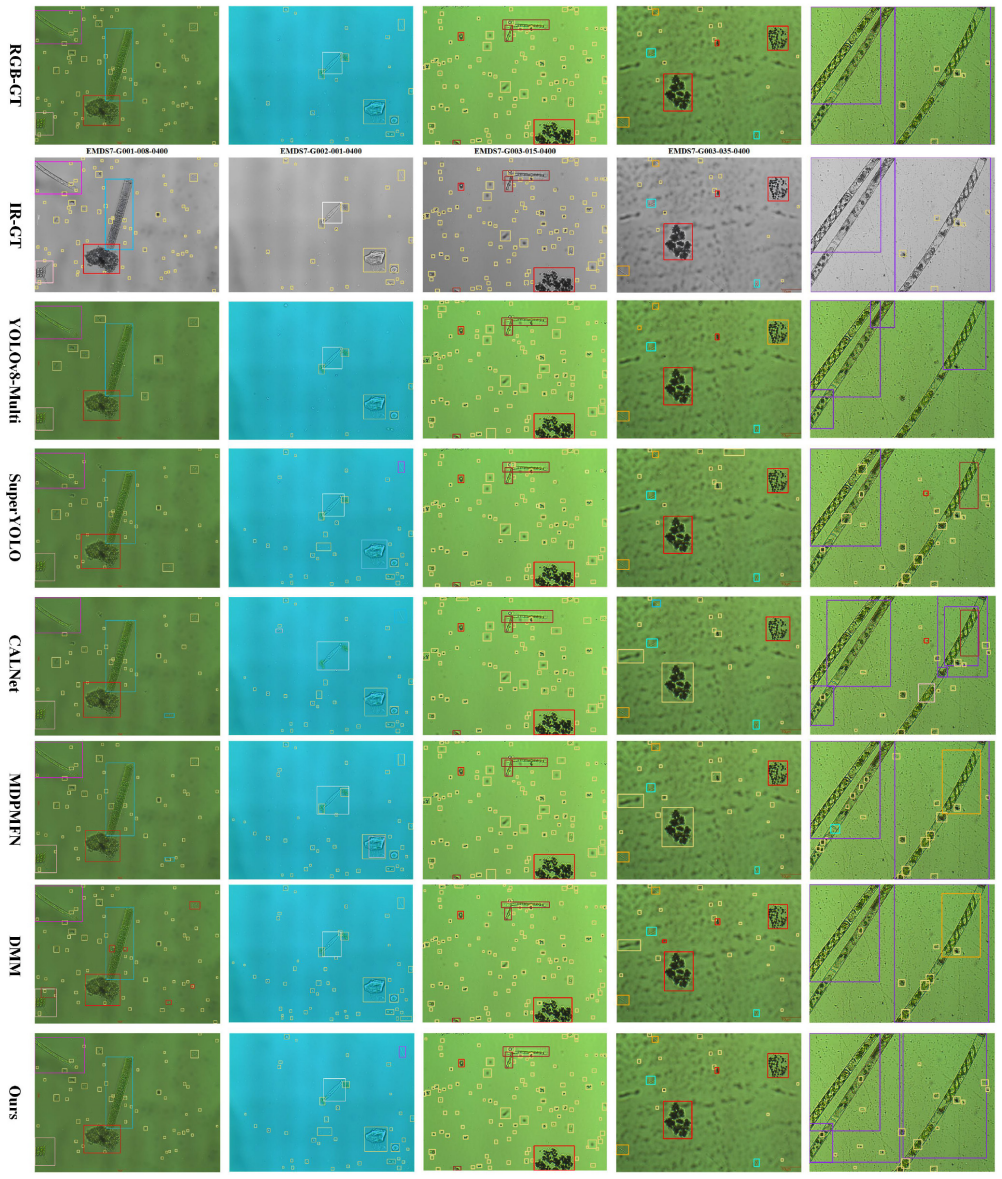
Visualization of representative multi-spectral microorganisms algae samples from the EMDS-7-MS dataset.

As shown in the Figure 7, the visualization results demonstrate that the proposed method significantly outperforms baseline models such as YOLOv8-Multi, CALNet, and DMM. Meanwhile, as observed from the first column of figures, the method proposed in this paper exhibits highly stable performance in multi-scale object detection, and achieves lower false detection and false positive rates compared with other methods. From the fourth column of detection results, it can be seen that Mamba-based methods yield a lower false positive rate in handling similar objects relative to other approaches. Furthermore, the proposed method outperforms other methods in detecting red-boxed objects. Finally, the fifth column of detection results demonstrates that our method also delivers excellent performance in large-scale object detection tasks. This confirms that when dealing with extremely small objects, high-aspect-ratio filaments, and cluttered backgrounds, the proposed method achieves superior localization accuracy and a lower miss rate, with predicted bounding boxes aligning most closely with the ground truth, thus validating its robustness in multi-scale feature extraction. Specifically, the wavelet fusion module enhances the discriminability of fine-grained texture features, which effectively reduces false positives for similar objects, while the Progressive Visual State Space (P-VSS) mechanism mitigates the miss rate of extremely small targets and high-aspect-ratio filaments by capturing long-range contextual dependencies across scales.

## 5 Conclusion

To address the challenges of cross-band spectral heterogeneity, small object size, and high visual similarity in multi-spectral detection of environmental microorganisms, this paper proposes a fine-grained detection framework named WPF-Mamba. The framework enhances the capture of subtle morphological features through an innovative P-VSS Block (integrated with PMFF units), while simultaneously fusing multi-band complementary information using theWMF module via wavelet decomposition and frequency-domain alignment strategies. Experimental results on the extended EMDS-7-MS dataset demonstrate superior performance. By synergistically designing wavelet frequency-domain fusion and progressive feature refinement, the proposed framework constructs a robust and generalizable detection model, providing an effective paradigm for multi-spectral fine-grained microorganisms analysis. This work offers reliable technical support for environmental monitoring and ecological risk assessment, with potential for further expansion into practical application scenarios and structural optimization for lightweight, portable deployment.

## Data Availability

The raw data supporting the conclusions of this article will be made available by the authors, without undue reservation.
